# Repeated COVID-19 Vaccination as a Poor Prognostic Factor in Pancreatic Cancer: A Retrospective, Single-Center Cohort Study

**DOI:** 10.3390/cancers17122006

**Published:** 2025-06-16

**Authors:** Makoto Abue, Mai Mochizuki, Rie Shibuya-Takahashi, Kensuke Ota, Yuta Wakui, Wataru Iwai, Jun Kusaka, Masashi Saito, Shinichi Suzuki, Ikuro Sato, Keiichi Tamai

**Affiliations:** 1 Departments of Gastroenterology, Miyagi Cancer Center, Natori 981-1293, Miyagi, Japan; 2 Divisions of Cancer Stem Cell, Miyagi Cancer Center Research Institute, Natori 981-1293, Miyagi, Japan; 3 Department of Pathology, Miyagi Cancer Center, Natori 981-1293, Miyagi, Japan

**Keywords:** pancreatic cancer, COVID-19 vaccination, mRNA vaccine, IgG4, cancer immunity

## Abstract

**Background/Objectives**: The COVID-19 vaccine is a significant technological advancement with widespread global use. However, its effect on cancer immunity, particularly with repeated vaccinations, remains unclear. We aimed to investigate the relationship between repeated vaccinations and pancreatic cancer (PC) prognosis. Additionally, we examined serum IgG4 levels, known to be an immune suppressor which increases with repeated vaccinations. **Methods**: We retrospectively examined the effect of vaccination on survival in 272 PC patients diagnosed at our hospital from January 2018 to November 2023 and analyzed prognostic factors, including IgG4 levels in 96 PC patients. Immunohistochemistry for Foxp3 in the tumor tissue was performed, and the serum IgG4 level was measured. Serum samples from 79 patients with benign and malignant diseases, including PC, were collected between September and November 2023, and the spike-specific IgG4 level was determined using an enzyme-linked immunosorbent assay. **Results**: The overall survival (OS) of PC patients was shortened in those vaccinated three times or more, and the total serum IgG4 levels increased with the number of vaccinations. Of note, OS was significantly shorter in the high IgG4 group, and Foxp3-positive cells in the tumor tissues were increased. Repeated vaccinations increased the spike-specific IgG4 levels, and a positive correlation was observed between spike-specific IgG4 and the total IgG4. **Conclusions**: These findings highlight repeated vaccination as a poor prognostic factor in PC patients and suggest that IgG4 is induced by repeated vaccination and may be associated with a poor prognosis in these patients.

## Simple Summary

Repeated COVID-19 vaccination is known to increase spike-specific immunoglobulin G4 (IgG4), and there are concerns regarding its impact on cancer immunity. This study aimed to investigate the relationship between repeated COVID-19 vaccination and prognosis in patients with pancreatic cancer (PC). The study findings were that repeated COVID-19 booster vaccinations are associated with poorer overall survival in patients with PC. Notably, our analysis reveals that high levels of IgG4, induced by vaccination, correlate with a detrimental prognosis in these patients. These insights provide essential information regarding the interplay between vaccination and cancer progression, which has significant implications for patient management strategies. Our study highlights the necessity for ongoing research into the long-term effects of mRNA vaccinations on cancer prognosis, which remains a pressing concern in the evolving landscape of public health and cancer treatment.

## 1. Introduction

Pancreatic cancer (PC) is an intractable cancer with a poor prognosis, the third leading cause of cancer-related deaths in the United States, and the fourth leading cause of cancer-related deaths in Japan [1,2]. In recent years, surveillance based on known risk factors (e.g., family history, diabetes mellitus, intraductal papillary mucinous neoplasm, chronic pancreatitis, and obesity) and the identification of the imaging features of early PC [3,4,5,6,7,8,9,10,11,12,13,14] have facilitated early detection and an improved prognosis [15,16,17,18,19]. Improved chemotherapy [20,21,22] and adjuvant therapy in combination with surgery have also improved prognoses [23,24,25,26].

Severe acute respiratory syndrome coronavirus 2 (SARS-CoV-2) infection began to spread in December 2019 and eventually became a pandemic [27]. The mRNA vaccines against SARS-CoV-2 have played a major role in controlling the coronavirus disease 2019 (COVID-19) pandemic [28]. In Japan, two mRNA vaccines, i.e., BNT162b2 (Pfizer-BioNTech, New York, NY, USA) and mRNA-1273 (Moderna, Cambridge, MA, USA), were approved in February and May 2021, respectively [29,30]. A booster vaccination (third vaccination dose) was initiated for healthcare workers in December 2021 and for the general population in January 2022. In Japan, the total number of vaccinations exceeds 400 million, with a two-dose vaccination rate of 79.8% and a three-dose vaccination rate of 67.3%, and the number of people who received more than four doses exceeds 130 million [31].

The mRNA vaccine is a new type of vaccine in which synthetic mRNA molecules containing the coding sequence necessary to construct the SARS-CoV-2 spike protein are encapsulated in lipid nanoparticles to enable mRNA delivery to cells [32,33], resulting in the production of SARS-CoV-2 spike antigens and the subsequent induction of neutralizing antibodies [34]. While immunoglobulin G1 (IgG1) is the main and most abundantly induced immune factor after vaccination [35,36], the IgG4 level also increases with repeated vaccination over a short period [37]. Prolonged exposure to the same antigen induces a class switch of B lymphocytes to produce IgG4, resulting in a decrease in fragment crystallizable (Fc) receptor-mediated effector functions, including antibody-dependent cell phagocytosis and antibody-dependent cytotoxicity against cancer, ultimately leading to the immune evasion of cancer [38,39]. In PC, the total serum IgG4 level is mildly elevated (<2-fold) in approximately 10% of the patients, with only 1% exceeding 280 mg/dL [40]. Additionally, a high degree of IgG4-positive plasma cell infiltration, as revealed by pathological examination after resection, is associated with a poor prognosis [41]. Several studies have reported the association of increased IgG4 levels with a poor prognosis in intrahepatic cholangiocarcinoma, extrahepatic cholangiocarcinoma, gastric cancer, and esophageal cancer, but not in PC [39,41,42,43,44]. Foxp3-positive regulatory T cells (Treg) also play an important role in the IgG4 response and may lead to a poor prognosis through the immune evasion of cancer [45,46].

Therefore, this study aimed to investigate the relationship between SARS-CoV-2 mRNA vaccination and the prognosis in patients with PC.

## 2. Materials and Methods

### 2.1. Patients

**Figure 1:**
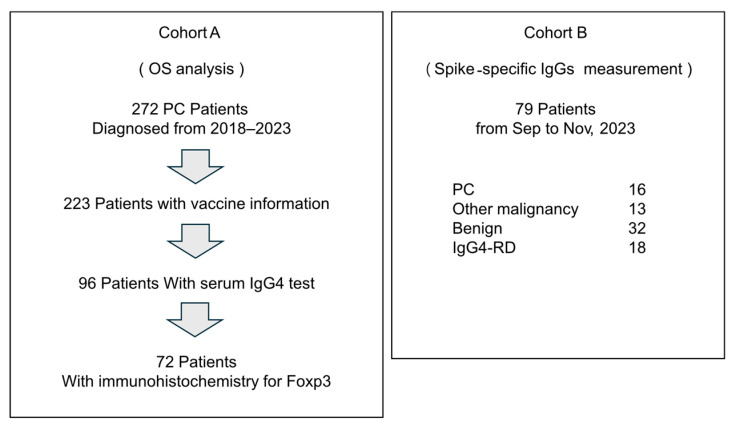
A schema of cohorts in this study.

We performed a retrospective analysis at the Department of Gastroenterology, Miyagi Cancer Center, where 272 patients with PCs were enrolled between January 2018 and November 2023 (Cohort A, Figure 1). Among them, the vaccination history information at diagnosis was available for 223 patients, and the total IgG and IgG4 levels were determined (turbidimetric and latex turbidimetric immunoassays, respectively; BML, Tokyo, Japan) in 96 patients. Of these 96 patients, biopsies or surgical resections, as well as immunohistochemistry for Foxp3, were performed in 72 patients. The specimens from the 72 cases comprised 30 surgical specimens, 39 endoscopic ultrasound-guided fine-needle aspiration biopsy (EUS-FNA) specimens, and 3 biopsy specimens of the duodenal and bile duct invasion sites, all of which were primary PC.

To measure the spike-specific antibodies, a further 79 patients [patients with PC (n = 16), cancer other than PC (n = 13), benign disease (n = 32), and IgG4-related disease (IgG4-RD) (n = 18)] at the Department of Gastroenterology, Miyagi Cancer Center, between September and November 2023, were registered prospectively (Cohort B, Figure 1). Blood samples were collected, and the total IgG, total IgG4, and spike-specific IgG levels were measured. Information on the number of COVID-19 vaccinations received by patients at the time of blood specimen collection was obtained from medical records. We collected tumor information on the pathological stage based on the UICC TNM classification of malignant tumors (8th edition). The Eastern Cooperative Oncology Group Performance Status (PS) Scale was used to assess the patient’s general condition at the initial visit.

### 2.2. Immunohistochemistry

Immunohistochemistry for Foxp3 was performed on 72 patients in Cohort A using the Ventana Benchmark system (Roche, Basel, Switzerland). The primary antibody used in this study was the anti-Foxp3 antibody (ab20034; Abcam).

### 2.3. Enzyme-Linked Immunosorbent Assay

Spike-specific IgG (total IgG, IgG1, IgG2, IgG3, and IgG4) levels were determined using an enzyme-linked immunosorbent assay (ELISA) in Cohort B, as described previously, with slight modifications [47]. Briefly, 96-well plates (#9018, Corning, Corning, NY, USA) were coated overnight at 4 °C with SARS-CoV-2 full-length recombinant spike protein (1 μg/mL in bicarbonate buffer [pH 9.8], R&D Systems, Minneapolis, MN, USA). For calibration, 96-well plates were coated overnight at 4 °C with purified human IgG1 (ab90283, Abcam, Cambridge, UK, 125 to 8000 ng/mL in bicarbonate buffer [pH 9.8]), IgG2 (ab90284, Abcam, 62.5 to 4000 ng/mL), IgG3 (ab118426, Abcam, 15.63 to 1000 ng/mL), IgG4 (ab183266, Abcam, 31.25 to 2000 ng/mL), and total IgG (143-09501, Fujifilm Wako, Osaka, Japan, 7.81 to 500 ng/ml). After washing with phosphate-buffered saline containing 0.1% Tween20 (PBS-T), the plates were blocked with PBS-T containing 1% BSA. Next, serum samples were added to the plates at dilutions between 1:10 and 1:10,000, followed by incubation at 32 °C for 1 h. Antigen-bound antibodies were then detected with HRP-conjugated mouse anti-human subclass specific antibodies (IgG1: 9054-05, IgG2: 9060-05, IgG3: 9210-05, IgG4 9200-05, and total IgG: 2040-05, Southern Biotech, Birmingham, AL, USA). The absorbance was measured at 450 nm with a reference wavelength of 620 nm using a plate reader (Synergy LX; Agilent, Santa Clara, CA, USA).

### 2.4. Statistics Analysis

Statistical analyses were performed using GraphPad Prism (GraphPad Software v10.1.2(324), San Diego, CA, USA). Non-parametric statistical tests, such as the Mann–Whitney test for two groups and the Kruskal–Wallis test with Dunn’s multiple comparisons test for multiple independent groups, were used. Survival curves were generated using the Kaplan–Meier method, and the log-rank test or Gehan-Breslow-Wilcoxon test was used for significance testing. In addition, a propensity score matching method was used to reduce the effects of confounding. Matching was performed using R software (4.2.2) with a caliper width equal to 0.2 of the standard deviation of the log of the propensity score, nearest neighbor method, and 1:1 matching protocol without replacement. Variables were selected on the basis of clinical experience, and the success of balancing distributions between two groups. Multivariate analysis of the prognostic factors, including neutrophil-to-lymphocyte ratio (NLR) [48], modified Glasgow prognostic score (mGPS) [49], and Prognostic Nutritional Index (PNI) [50], were performed using Cox proportional hazards analysis. The mGPS and PNI were defined as according to previous reports [49,50] as follows: C-reactive protein (CRP) level ≤ 1.0 mg/dL and Alb level ≥ 3.5 g/dL; score 0; CRP level > 1.0 mg/dL or Alb level < 3.5 g/dL; score 1; CRP level > 1.0 mg/dL and Alb level < 3.5 g/dL; score 2. PNI = 10 × Alb (g/dL) + 0.005 × lymphocyte (/µL)). The cutoff values for NLR, PNI, and IgG4 were determined by checking the area under the curve (AUC) using the SurvivalROC package in R software. The data were then binarized using the 2-year predicted survival rate that showed the best AUC. We determined the cutoff value with the smallest distance from the upper left corner of the ROC curve drawn by the pROC package (Supplementary Figure S1). Foxp3-positive cells in immunohistochemistry were observed in a 40x higher power field of view, and the number of Foxp3-positive cells in the total cells within 0.01 square millimeter (100 μm × 100 μm) was counted in five areas using NDP.view2 image viewing software (U12388-01, HAMAMATSU). Hematoxylin-positive cells were counted for total cell counts. Scatter plots of spike-specific IgG4 and total IgG4 were created, and regression lines along with coefficients of determination (R^2^) were calculated using R software (4.2.2). Statistical significance was set at * *p* < 0.05.

## 3. Results

### 3.1. Repeated COVID-19 Vaccination Worsens the Prognosis of PC Patients

**Figure 2:**
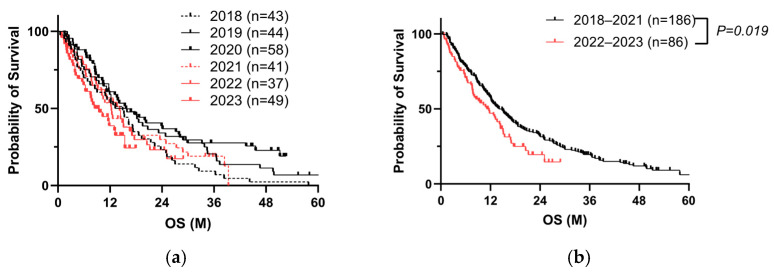

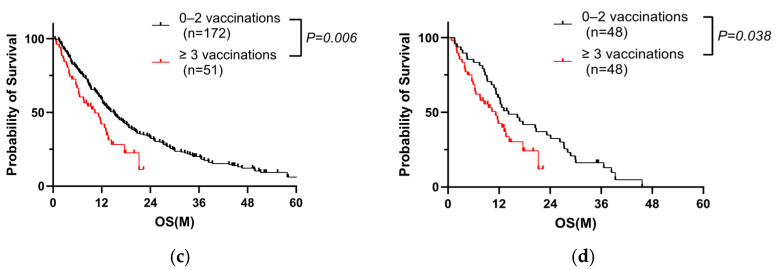
Repeated COVID-19 vaccination correlates with poor prognosis of PC. (**a**) Kaplan–Meier analysis from 2018 to 2023 in Cohort A by year. (**b**) Kaplan–Meier analysis of 272 PC patients in Cohort A (log-rank test, *p* = 0.019, median 11.2 months vs. median 14.1 months). (**c**) Kaplan–Meier analysis of 223 PC patients with known vaccination history in Cohort A (log-rank test, *p* = 0.006, median 10.3 months vs. median 14.9 months). (**d**) Kaplan–Meier analysis of 96 PC patients after propensity score matching for TNM factors, surgery, and chemotherapy in Cohort A (log-rank test, *p* = 0.038, median 11.2 months vs. median 14.2 months).

We analyzed the prognosis of PC patients before and after receiving SARS-CoV-2 vaccination from 2018 to 2023 (Table 1, Cohort A). Patient outcomes had improved each year by 2020; however, it began to deteriorate in 2021 (Figure 2a). The outcomes in 2022–2023 were significantly worse than those in 2018–2021 (Figure 2b). Cox proportional hazards analysis indicated that a history of receiving COVID-19 vaccinations (three or more times) at the initial visit, PS, jaundice, high TNM factors, no surgery, no chemotherapy, and high tumor markers including carcinoembryonic antigen (CEA) and carbohydrate antigen (CA) 19-9 significantly affected the overall survival (OS) (Table 2). Of 272 cases, the information of vaccination was recorded in 223 cases. When divided into two groups, 0–2 vaccinations, or more than three vaccinations, we found a poorer prognosis in the latter (Figure 2c). After propensity score matching for TNM factors, surgery, and chemotherapy, we obtained a similar result (Figure 2d, Table 3). These data suggest that repeated vaccinations are a negative prognostic factor.

### 3.2. High Total IgG4 Level Correlates with a Poor PC Prognosis

**Figure 3:**
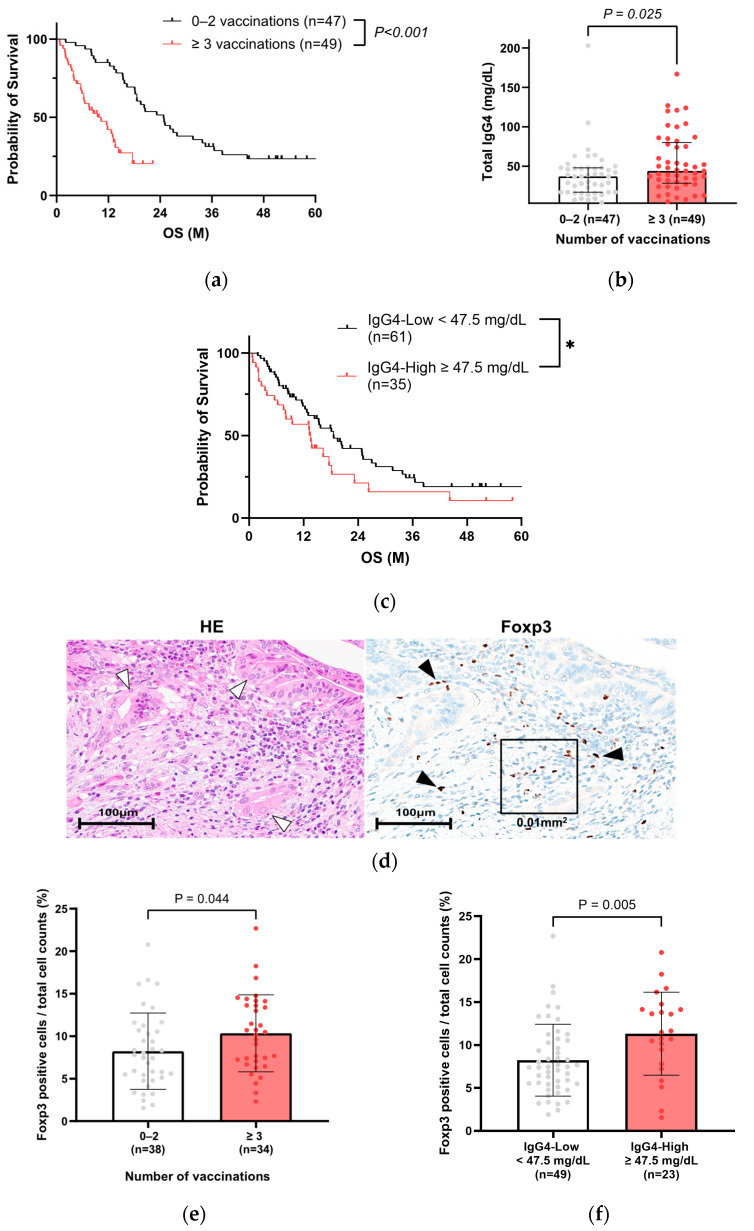
IgG4 is a negative prognostic factor in PC patients. (**a**) Kaplan–Meier analysis of 96 PC patients with known vaccination history and measured IgG4 levels in Cohort A (log-rank test, p < 0.001, median 10.3 months vs. median 20.8 months). (**b**) Comparison of total IgG4 levels by number of vaccinations in 96 PC patients of Cohort A (Mann–Whitney test, *p* = 0.025, ≥3 vaccinations vs. 0–2 vaccinations). (**c**) Kaplan–Meier analysis in PC patients with IgG4 test in Cohort A (* log-rank test, *p* = 0.076 and Gehan-Breslow-Wilcoxon test, *p* = 0.042, IgG4-high group vs. IgG4-low group, cutoff value for IgG4 is 47.5 mg/dL). (**d**) Representative images of PC tissues (white arrowheads: tumor cells) with Foxp3-positive lymphocytes (black arrows). A square frame (100 µm × 100 µm) was used to measure the number of Foxp3-positive cells/total cell counts. (**e**) Comparison of Foxp3-positive cells/total cell counts by number of vaccinations (Mann–Whitney test, *p* = 0.044, ≥3 vaccinations vs. 0–2 vaccinations). (**f**) Comparison of Foxp3 positive cells/total cell counts between high and low serum IgG4 groups (Mann–Whitney test, *p* = 0.005, cutoff value for IgG4 is 47.5 mg/dL). PC, pancreatic cancer.

We hypothesized that IgG4, known to be induced by COVID-19 vaccination, deteriorates the prognosis of PC patients. Therefore, we compared the relationship between IgG4 levels and vaccination. Of 223 cases, serum IgG4 value was recorded in 96 patients with vaccination history information (Table 3). In this population, the three or more vaccination group also had a poorer prognosis (Figure 3a), similar to Figure 2c. The characteristics of the 96 PC patients in Cohort A are shown in Table 4. The group with three or more vaccinations had a significantly higher NLR and lower PNI, which were also extracted as significant factors in the multivariate analysis (Supplementary Table S1). The same result was obtained when 66 patients with matched backgrounds were examined, excluding surgical cases (Supplementary Figure S2, Tables S2 and S3). The total IgG4 levels were significantly higher in the three or more vaccination group (Figure 3b, Table 4), particularly for five or more vaccinations (Supplementary Figure S3). When the patients were divided into two groups based on the total IgG4 expression (Supplementary Table S4), the prognosis was significantly worse in the IgG4-high group (Figure 3c). When the patients were divided into two groups based on the OS, the total IgG4 level was also significantly higher in the short OS (<90 days) group (Supplementary Figure S4).

To investigate the relationships between IgG4 and Tregs in patients who have been repeatedly vaccinated, immunohistochemical analysis of Foxp3 was performed in 72 of 96 cases who had undergone surgical resection or endoscopic biopsy (Supplementary Table S5). Foxp3-positive cells were observed around tumor cells (Figure 3d). The average percentage of Foxp3-positive cells in the total cell counts was a median of 8.3 ± 4.6% in 72 cases, and the percentage of Foxp3-positive cells identified in or around the tumor cells was significantly higher in the three or more vaccination group (Figure 3e) and in the group with high serum IgG4 levels (Figure 3f).

### 3.3. Total IgG4, Along with Spike-Specific IgG4, Is Increased in Patients with Repeated COVID-19 Vaccination

**Figure 4:**
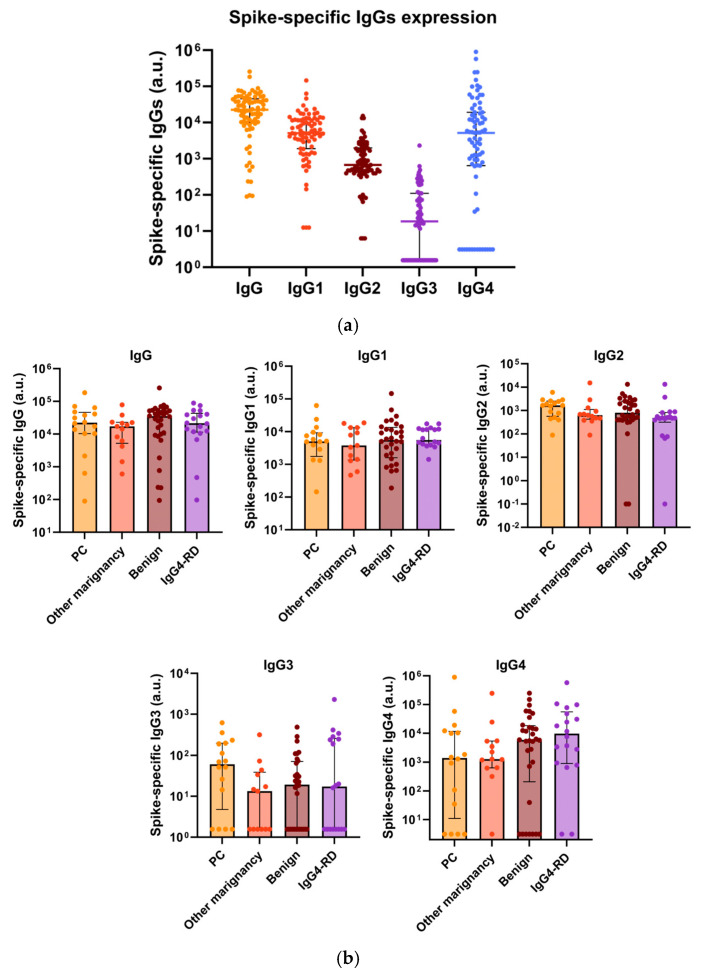

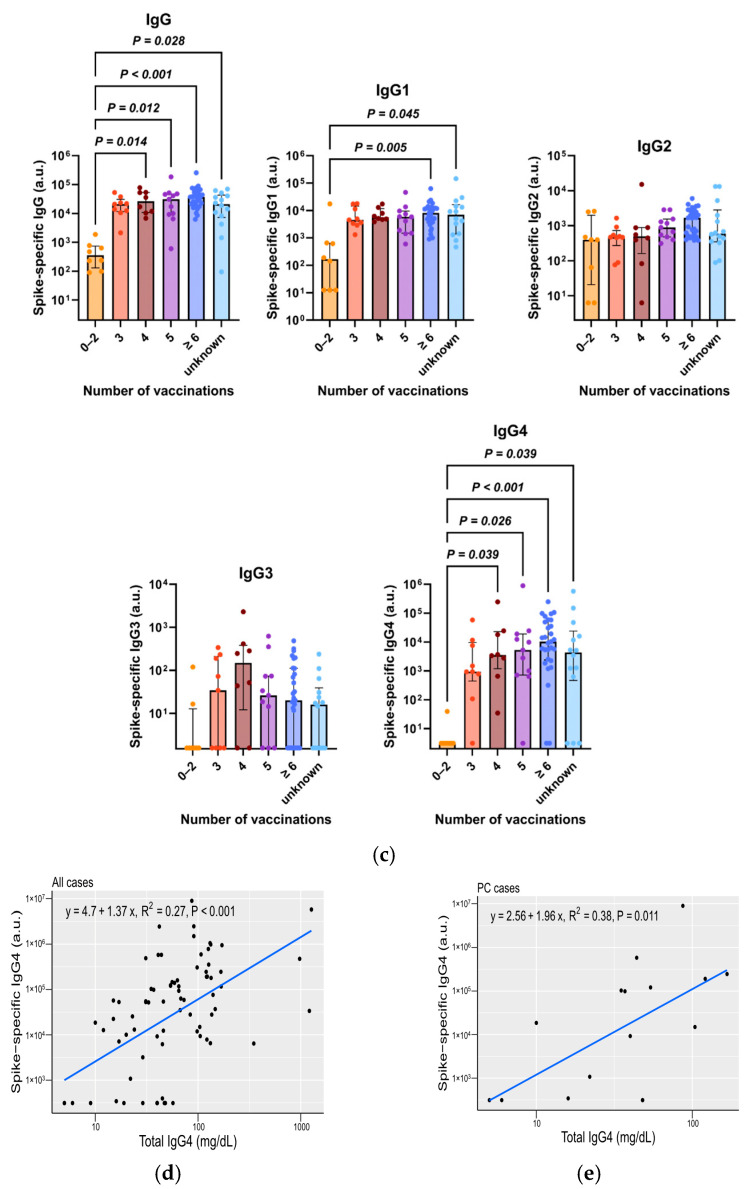
Total IgG4, along with spike-specific IgG4, is increased in patients with repeated COVID-19 vaccination. (**a**) Measurement of spike-specific IgGs in Cohort B using ELISA. (**b**) Comparison of spike-specific IgGs values between types of diseases. There was no significant difference in the spike-specific IgGs levels between the disease types. (**c**) Comparison of spike-specific IgGs values based on the numbers of vaccinations. Spike-specific IgG, IgG1, and IgG4 levels increased in the groups that were vaccinated more than three times (Kruskal–Wallis test, *p* < 0.05, vs. 0–2 vaccinations). (**d**,**e**) Correlation plot of the total IgG4 and spike-specific IgG4 in all cases (R^2^ = 0.27, *p* < 0.001) (**d**) and PC cases (R^2^ = 0.38, *p* = 0.011). (**e**) ELISA, enzyme-linked immunosorbent assay; PC, pancreatic cancer.

To confirm that the increase in the total IgG4 level was due to spike-specific IgG4, we determined the total and spike-specific IgGs in Cohort B. The characteristics of 79 patients in Cohort B are shown in Supplementary Table S4. We included malignant and benign diseases other than PC to determine whether the changes in IgGs depend on specific diseases. Spike-specific IgG1 and IgG4 were detected in higher amounts, whereas IgG2 and IgG3 were detected in lower amounts (Figure 4a). No significant difference was noted in the spike-specific IgG levels, including IgG4, between the disease types (Figure 4b). Spike-specific IgG4, IgG1, and IgG levels increased in the groups that were vaccinated more than three times (Figure 4c). Total IgG4 and spike-specific IgG4 levels were positively correlated in both cases (Figure 4d) and PC patients (Figure 4e). The spike-specific IgG4 high group tends to be a poorer prognosis than the spike-specific IgG4 low group in PC cases, although the difference is not significant due to the small samples (n = 16) (Supplementary Figure S5).

## 4. Discussion

In Japan, the mRNA-type COVID-19 vaccine is mainly used for initial immunization, and additional immunizations are repeatedly administered to prevent severe disease [31]. However, repeated immunization with the COVID-19 vaccine can accelerate the transition to IgG4 [51] and increase spike-specific IgG4 levels [37,38,51], which is consistent with our findings. Elevated IgG4 levels can promote cancer growth by suppressing cancer immunity and is associated with a poor prognosis [39,41,42,43,44]. Only a few countries administer more than five doses of vaccinations, and the impact of repeated vaccinations on IgG4 levels against cancer are unclear. In this study, our results demonstrated that more than three vaccination doses correlated with a poor prognosis in patients with PC, particularly in those whom the total IgG4 level was increased after vaccination. A higher total IgG4 level also correlated with a poor prognosis. The Gehan-Breslow-Wilcoxon test showed a marked difference and has a particularly large impact on early mortality. IgG4 was elevated in patients who had an early course of 90 days or less. There was an overall positive correlation between total IgG4 and spike-specific IgG4 levels. Our findings collectively indicate that spike-specific IgG4 could be correlated to the prognosis of PC patients. To the best of our knowledge, this is the first study to report a correlation between SARS-CoV-2 vaccination and PC prognosis.

In this study, high NLR and mGPS1-2, and low PNI, all of which are nutritional indices and indicators of a poor prognosis in PC [48,49,50,52], were significantly correlated with a poor prognosis. Multivariate analysis revealed that repeated vaccination, but not NLR, mGPS, and PNI, was an independent poor prognostic factor, suggesting that the nutritional indices are confounding factors. Indeed, repeated vaccination was significantly correlated with NLR and PNI. A previous report also demonstrated that vaccination increases NLR [53]. These data suggest that vaccination influences neutrophils and lymphocytes. Further study should elucidate whether vaccinations alter leukocyte subsets, resulting in an increase in IgG4 production.

Vaccination also alters other immune system components, beside IgG4, and potential side effects have been reported in previous preclinical studies. Seneff et al. reported that vaccination suppresses type I interferon signaling and has various adverse effects on human health, including cancer surveillance [54]. BNT162b2 vaccination led to an increase in Tregs [55,56]. Tregs, a subset of CD4+T cells expressing Foxp3, play critical roles in suppressing immune responses and migrating toward tumors in the presence of chemokines to suppress antitumor immune responses, causing cancer cells to grow and proliferate [57]. A high infiltration by Tregs has been associated with poor survival in various types of cancer [58]. In mice, the percentage of CD25+Foxp3+CD4+ Tregs and the levels of immunosuppression cytokines IL-10 were up-regulated after extended RBD vaccine booster vaccination. This may result in the reduced activation and differentiation of B cells on antigen stimulation, functional inhibition of antigen-presenting cells, a consequential decrease in CD8+T cell activation, and increased PD-1 and LAG-3 expression in these T cells [59]. Repeated antigen exposure leads to T cell exhaustion [60,61,62].Collectively, repeat vaccination with the COVID-19 mRNA vaccine booster may be a potential risk for cancer. Some studies have reported the association between SARS-CoV-2 spike and p53 [63,64]. This study also showed a significant correlation between serum IgG4 levels and Foxp3-positive cell infiltration in tissues, suggesting that Treg could be a factor for a poor prognosis in repeatedly vaccinated patients. Further studies are needed to determine the detailed pathway through which repeated vaccinations affect the prognosis.

Previous studies indicate that the number of IgG4-positive plasma cells in the tissues increases in IgG4-RD, with elevated total serum IgG4 levels [65,66]. Increased IgG4-positive plasma cell infiltration into or around cancer tissues has been associated with a poor prognosis in cancers including PC, cholangiocarcinoma, gastric cancer, and esophageal cancer [39,41,42,43,44]. The high-level intratumoral infiltration of IgG4-positive plasma cells is an independent predictor for poor OS in PC patients after curative resection, and M2-polarized tumor-associated macrophages within the tumor are associated with IgG4 induction [41]. In cholangiocellular carcinoma, the involvement of Treg and IL-10 in IgG4 induction has also been reported [43]. Moreover, elevated total serum IgG4 levels, regardless of antigen specificity, may affect the cancer microenvironment [39]. Repeated COVID-19 mRNA vaccination results in IgG4 class switching and decreased NK cell activation by S1-specific antibodies [67,68]. Locally increased IgG4 in the cancer microenvironment should inhibit antibody-mediated anticancer responses, allowing cancer to evade a local immune attack and indirectly promote cancer growth; moreover, higher levels of IgG4 have been associated with more aggressive cancer progression [39]. It should be noted, however, that the effect of elevated IgG4 titers on Fc function may depend on the interplay between the antibody and antigen [68]. Identifying spike-specific IgG4 in tumor tissues would facilitate future analysis of the relationship between vaccination and prognosis.

## 5. Limitations

This study was a retrospective, single-center cohort study comprising 96 cases of PC. However, the 79 cases in which anti-spike IgG4 was measured included specimens that were collected in approximately 3 months. The sample size of 96 cases was small, corresponding to 96 out of 272 PC patients treated during the same period, and all the PC cases with IgG4 were not measured. Therefore, there is a possibility of bias in the sample selected for IgG4 measurements. All the stages of PC were included in this study. The number of vaccination doses considered in this study did not account for subsequent vaccinations received after the blood collection or history of COVID-19. The study also did not consider potential confounding factors such as patient comorbidities, concurrent treatment, or vaccine type. The present study revealed discrepancies between the Gehan-Breslow-Wilcoxon test results and the multivariate analysis results regarding the effect of IgG4 on PC prognosis. Because of the small sample size, it is difficult to explain this discrepancy precisely. Further study is required to determine the impact of IgG4 on PC prognosis. Future research should incorporate a larger cohort size and investigate additional mechanisms involved in the immunity to SARS-CoV-2 following vaccination.

## 6. Conclusions

Repeated vaccination is a poor prognostic factor in PC patients. Repeated vaccination increased the serum total and spike-specific IgG4 levels, which may be associated with a poor prognosis.

## Figures and Tables

**Table 1:** Characteristics of PC patients in Cohort A (n = 272).

	All Case (n = 272)	2018–2021 (n = 186)	2022–2023 (n = 86)	*p*-Value
Age (mean ± SD)	70.9 ± 9.6	70.9 ± 9.6	70.5 ± 9.9	n.s. ^a^
Age ≥ 75, no. (%)	103 (37.9)	71 (38.2)	32 (37.2)	n.s. ^b^
Female, no. (%)	133 (48.9)	92 (49.5)	41 (47.7)	n.s. ^b^
PS ≥ 2, no. (%)	42 (15.4)	27 (14.5)	15 (17.4)	n.s. ^b^
Jaundice, no. (%)	92 (33.8)	63 (33.9)	29 (33.7)	n.s. ^b^
Diabetes Mellites, no. (%)	144 (52.9)	97 (52.2)	47 (54.7)	n.s. ^b^
Location (head), no. (%)	126 (46.3)	90 (48.4)	36 (41.9)	n.s. ^b^
UICC TNM classification				
T (3–4), no. (%)	148 (54.4)	95 (51.1)	53 (61.6)	n.s. ^b^
N, no. (%)	144 (52.9)	97 (52.2)	47 (54.7)	n.s. ^b^
M, no. (%)	146 (53.7)	97 (52.2)	49 (57.0)	n.s. ^b^
Surgery, no. (%)	61 (22.4)	51 (27.4)	10 (11.6)	0.005 ^b^
Chemotherapy, no. (%)	201 (73.9)	140 (75.3)	61 (70.9)	n.s. ^b^
COVID-19 mRNA ≥3 vaccinations (Yes/No/unknown)	51/190/31	0/186/0	51/4/31	<0.001 ^b^
Number of vaccinations (3/4/5/6/7)	12/8/19/9/3	-	12/8/19/9/3	
CEA median (min–max) ng/mL	4.8 (0.6–2660.6)	4.6 (0.6–2205.1)	5.1 (0.6–2660.6)	n.s. ^a^
CEA ≥ 10, no. (%)	77 (28.3)	55 (29.6)	22 (25.6)	n.s. ^b^
CA19-9 median (min–max) U/mL	442.0 (0–150,000)	449.0 (0–150,000)	450.2 (0–150,000)	n.s. ^a^
CA19-9 ≥ 500, no. (%)	132 (48.5)	90 (48.4)	42 (48.8)	n.s. ^b^

^a^ Mann–Whitney test. ^b^ Fisher’s exact test. PC, pancreatic cancer. PS, performance status. CEA, carcinoembryonic antigen. CA19-9, carbohydrate antigen 19-9. n.s., not significant.

**Table 2:** Cox proportional hazards analysis of factors affecting the prognosis of PC (n = 272).

	HR	95%CI	*p*-Value (Univariate)
Age ≥ 75	0.94	(0.71–1.23)	n.s.
Sex (Female/Male)	1.15	(0.88–1.50)	n.s.
PS ≥ 2	2.66	(1.85–3.74)	<0.001
Jaundice (Yes/No)	1.49	(1.12–1.97)	0.005
Diabetes Mellites (Yes/No)	0.77	(0.59–1.00)	n.s.
Location (head/body-tail)	0.95	(0.73–1.24)	n.s.
UICC TNM classification			
T (3–4/1–2)	2.31	(1.76–3.05)	<0.001
N (Yes/No)	1.57	(1.22–2.00)	<0.001
M (Yes/No)	3.88	(2.92–5.19)	<0.001
Surgery (Yes/No)	0.19	(0.13–0.28)	<0.001
Chemotherapy (Yes/No)	0.58	(0.43–0.79)	<0.001
COVID-19 mRNA ≥3 vaccinations (Yes/No)	1.72	(1.15–2.51)	0.006
CEA > 10 (ng/mL)	2.06	(1.54–2.73)	<0.001
CA19-9 > 500 (U/mL)	2.04	(1.56–2.69)	<0.001

PC, pancreatic cancer. PS, performance status. CEA, carcinoembryonic antigen. CA19-9, carbohydrate antigen 19-9. n.s., not significant.

**Table 3:** Characteristics of 223 PC patients with vaccination history information in Cohort A.

	Before Matching			After Matching		
	0–2 Vaccinations (n = 172)	≥3 Vaccinations (n = 51)	*p*-Value	0–2 Vaccinations (n = 48)	≥3 Vaccinations (n = 48)	*p*-Value
Age (mean ± SD)	70.6 ± 9.7	71.4 ± 9.3	n.s. ^a^	69.8 ± 9.5	71.4 ± 9.2	n.s. ^a^
Age ≥ 75, no. (%)	65 (37.8)	23 (45.1)	n.s. ^b^	16 (33.3)	21 (43.8)	n.s. ^b^
Female, no. (%)	84 (48.8)	25 (49.0)	n.s. ^b^	24 (50.0)	23 (47.9)	n.s. ^b^
PS ≥ 2, no. (%)	20 (11.6)	11 (21.6)	n.s. ^b^	8 (16.7)	11 (22.9)	n.s. ^b^
Jaundice, no. (%)	60 (34.9)	21 (41.2)	n.s. ^b^	15 (31.3)	18 (37.5)	n.s. ^b^
Diabetes Mellites, no. (%)	84 (48.8)	29 (56.9)	n.s. ^b^	28 (58.3)	28 (58.3)	n.s. ^b^
Location (head), no. (%)	87 (50.6)	24 (47.1)	n.s. ^b^	22 (45.8)	21 (43.8)	n.s. ^b^
UICC TNM classification						
T (3–4), no. (%)	85 (49.4)	33 (64.7)	n.s. ^b^	30 (62.5)	30 (62.5)	n.s. ^b^
N, no. (%)	90 (52.3)	30 (58.8)	n.s. ^b^	29 (60.4)	29 (60.4)	n.s. ^b^
M, no. (%)	89 (51.7)	30 (58.8)	n.s. ^b^	28 (58.3)	28 (58.3)	n.s. ^b^
Surgery, no. (%)	48 (27.9)	7 (13.7)	0.043 ^b^	7 (14.6)	7 (14.6)	n.s. ^b^
Chemotherapy, no. (%)	128 (74.4)	33 (64.7)	n.s. ^b^	33 (68.8)	33 (68.8)	n.s. ^b^
CEA ≥ 10, no. (%)	50 (29.1)	10 (19.6)	n.s. ^b^	16 (33.3)	9 (18.8)	n.s. ^b^
CA19-9 ≥ 500, no. (%)	82 (47.7)	25 (49.0)	n.s. ^b^	26 (54.2)	22 (45.8)	n.s. ^b^
IgG4 measurement, no. (%)	47 (27.3)	49 (96.1)	<0.001 ^b^	11 (22.9)	46 (93.9)	<0.001 ^b^
IgG4 (mean ± SD)	38.6 ± 31.7	54.7 ± 37.0	0.025 ^a^	44.2 ± 16.5	53.6 ± 36.8	n.s. ^a^

^a^ Mann–Whitney test. ^b^ Fisher’s exact test. PC, pancreatic cancer. PS, performance status. CEA, carcinoembryonic antigen. CA19-9, carbohydrate antigen 19-9. n.s., not significant.

**Table 4:** Characteristics of PC patients in Cohort A (n = 96).

	Patients (n = 96)	0–2 Vaccinations (n = 47)	≥3 Vaccination (n = 49)	*p*-Value
Age (mean ± SD)	71.4 ± 8.2	70.9 ± 9.6	70.9 ± 9.6	n.s. ^a^
Age ≥ 75, no. (%)	37 (38.5)	15 (31.9)	22 (44.9)	n.s. ^b^
Female, no. (%)	50 (52.1)	26 (55.3)	24 (49.0)	n.s. ^b^
PS ≥ 2, no. (%)	14 (14.6)	4 (8.5)	10 (20.4)	n.s. ^b^
Jaundice, no. (%)	40 (41.7)	19 (40.4)	21 (42.9)	n.s. ^b^
Diabetes Mellites, no. (%)	53 (55.2)	25 (53.2)	28 (57.1)	n.s. ^b^
Location (head), no. (%)	55 (57.3)	31 (66.0)	24 (49.0)	n.s. ^b^
UICC TNM classification				
T (3–4), no. (%)	44 (45.8)	13 (27.7)	31 (63.3)	<0.001 ^b^
N, no. (%)	47 (49.0)	18 (38.3)	29 (59.2)	0.045 ^b^
M, no. (%)	41 (42.7)	12 (25.5)	29 (59.2)	0.001 ^b^
Surgery, no. (%)	30 (31.3)	23 (48.9)	7 (14.3)	<0.001 ^b^
Chemotherapy, no. (%)	67 (69.8)	36 (76.6)	31 (63.3)	n.s. ^b^
CEA ≥ 10, no. (%)	15 (15.6)	6 (12.8)	9 (18.4)	n.s. ^b^
CA19-9 ≥ 500, no. (%)	40 (41.7)	15 (31.9)	25 (51.0)	n.s. ^b^
Other factors				
NLR (mean ± SD)	3.8 ± 2.5	3.0 ± 1.2	4.5 ± 3.2	0.008 ^a^
NLR ≥ 2.8, no. (%)	57 (59.4)	21 (44.7)	36 (73.5)	0.007 ^b^
mGPS (0/1/2)	68/19/9	36/8/3	32/11/6	n.s. ^b^
mGPS ≥ 1, no. (%)	28 (29.2)	11 (23.4)	17 (34.7)	n.s. ^b^
PNI (mean ± SD)	45.7 ± 5.4	47.3 ± 5.2	44.2 ± 5.3	0.002 ^a^
PNI ≥ 46.8, no. (%)	41 (42.7)	28 (59.6)	13 (26.5)	0.002 ^b^
IgG4 (mean ± SD)	46.8 ± 35.3	38.6 ± 31.7	54.7 ± 37.0	0.025 ^a^
IgG4 ≥ 47.5 mg/dL, no. (%)	35 (36.5)	12 (25.5)	23 (46.9)	0.035 ^b^

^a^ Mann–Whitney test. ^b^ Fisher’s exact test. PC, pancreatic cancer. PS, performance status. CEA, carcinoembryonic antigen. CA19-9, carbohydrate antigen 19-9. NLR, neutrophil-to-lymphocyte ratio. mGPS, modified Glasgow prognostic score. PNI, prognostic nutritional index. n.s., not significant.

## Data Availability

All data generated or analyzed during this study are included in this published article and its Supplementary Information Files.

## Supplementary Materials

The following supporting information can be downloaded at: https://www.mdpi.com/article/10.3390/cancers17122006/s1, Figure S1: Cutoff values for NLR, PNI, and IgG4 were determined based on ROC; Figure S2: Kaplan–Meier analysis of 66 PC patients in Cohort A without surgical treatment (log-rank test, *p* < 0.001, median 7.5 vs. median 18.1); Figure S3: Total IgG4 measured in patients with pancreatic cancer in Cohort A; Figure S4: Comparison of the serum IgG4 values between overall survival ≥ 90 days and <90 days groups (Mann–Whitney test, *p* = 0.033); Figure S5: Kaplan-Meier analysis of 16 PC patients divided into high and low spike-specific IgG4 levels in cohort B (log-rank test, *p* = 0.12, median undefined vs. median 6.3); Table S1: Cox proportional hazards analysis of factors affecting the prognosis of PC (n = 96); Table S2: Characteristics of 66 PC patients corrected for the exclusion of surgical cases in Cohort A; Table S3: Cox proportional hazards analysis of factors affecting PC prognosis (non-surgical cases n = 66); Table S4: Characteristics of high and low IgG4 groups in Cohort A; Table S5: Characteristics of 72 PC patients who underwent immunohistochemistry in Cohort A; Table S6: Characteristics of 79 patients in Cohort B.

## Abbreviations

The following abbreviations are used in this manuscript:

PC	Pancreatic cancer
SARS-CoV-2	Severe acute respiratory syndrome coronavirus 2
COVID-19	Coronavirus disease 2019
IgG	Immunoglobulin G
PS	Performance status
CEA	Carcinoembryonic antigen
CA19-9	Carbohydrate Antigen 19-9
IgG4-RD	IgG4-related disease
ELISA	Enzyme-linked immunosorbent assay
NLR	Neutrophil-to-lymphocyte ratio
mGPS	modified Glasgow prognostic score
PNI	Prognostic Nutritional Index
CRP	C-Reactive Protein
AUC	Area Under the Curve.
ROC	Receiver Operating Characteristic
OS	Overall Survival
