# Supplementary material for: Repeated COVID-19 Vaccination as a Poor Prognostic Factor in Pancreatic Cancer: A Retrospective, Single-Center Cohort Study
Source: Cancers (Basel). 2025 Jun 16;17(12):2006. doi: 10.3390/cancers17122006 (PMC12191412; doi:10.3390/cancers17122006)
Supplement: Supplementary file 1 [file cancers-17-02006-s001.zip › cancers-3615417-supplementary.pdf]

Supplementary Material

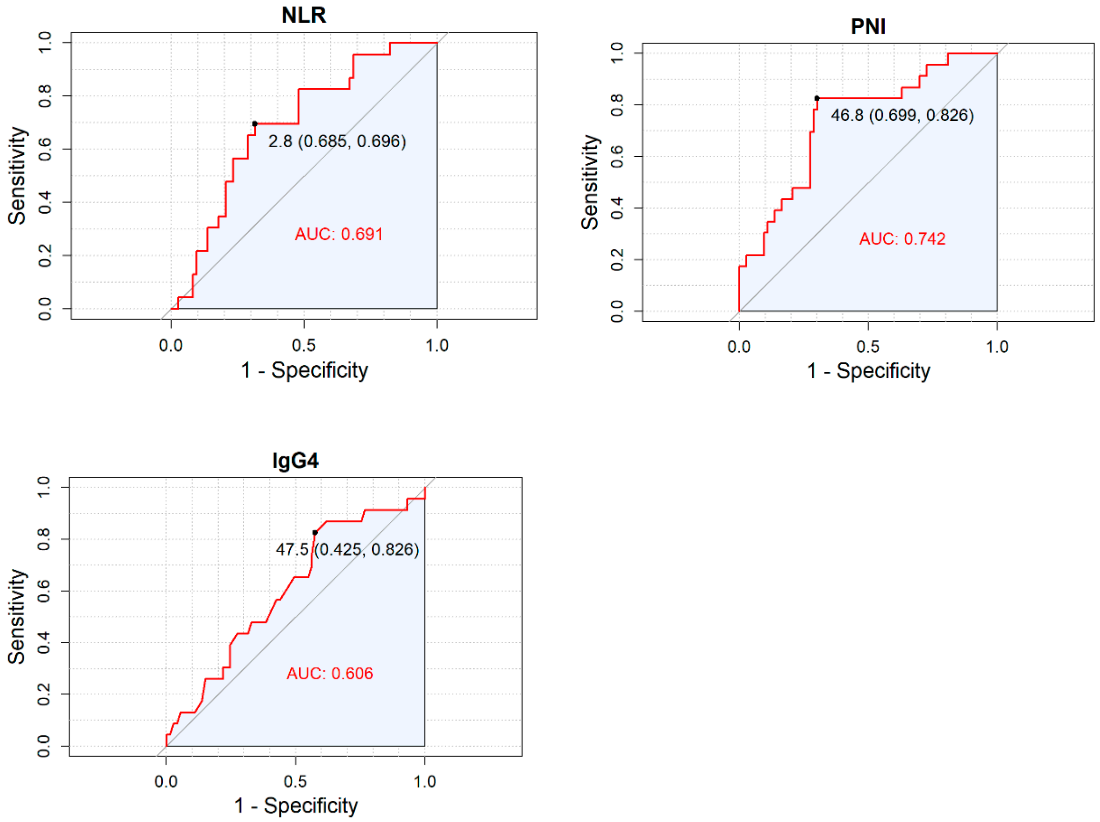

Figure S1. Cutoff values for NLR, PNI, and IgG4 were determined based on ROC. The cutoff values (specificity and sensitivity) are indicated by the black dots on the ROC curve. NLR, Neutrophil-to-lymphocyte ratio; PNI, Prognostic Nutritional Index; ROC, receiver operating characteristic. AUC, Area Under the Curve.

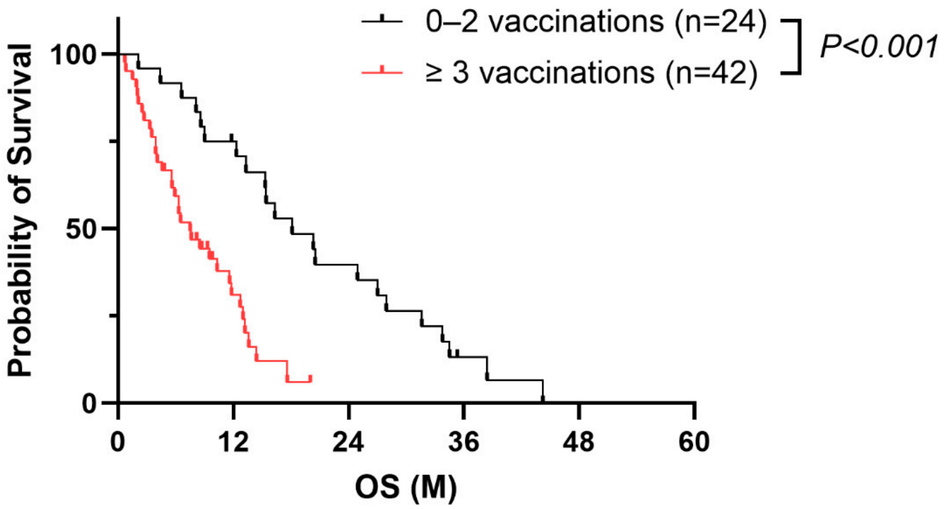

Figure S2. Kaplan–Meier analysis of 66 PC patients in Cohort A without surgical treatment (log-rank test, $p < 0.001$, median 7.5 vs. median 18.1). PC, pancreatic cancer.

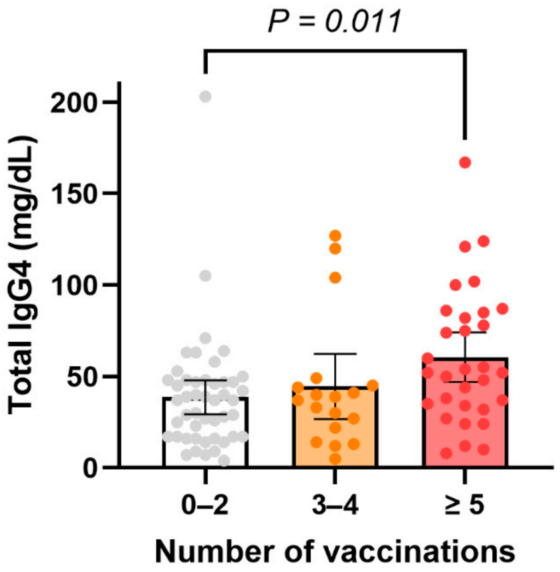

Figure S3. Total IgG4 measured in patients with pancreatic cancer in Cohort A. Serum total IgG4 levels are significantly increased with ≥ 5 vaccinations, compared with 0–2 vaccinations (Kruskal–Wallis test, $p = 0.011$).

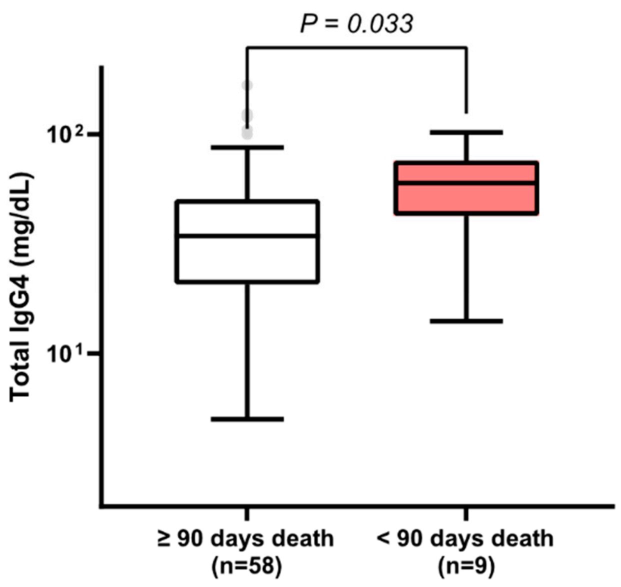

Figure S4. Comparison of the serum IgG4 values between overall survival ≥ 90 days and < 90 days groups (Mann–Whitney test, $p = 0.033$).

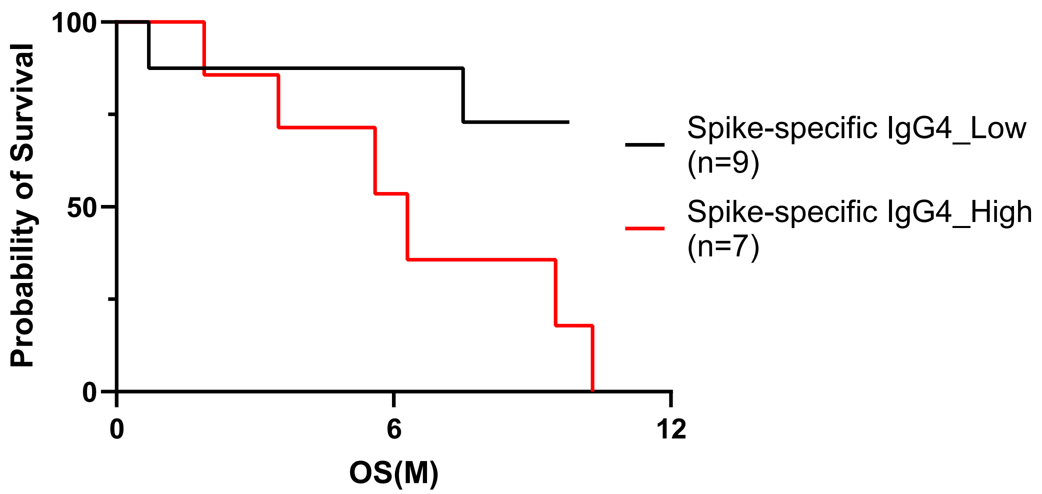

Figure S5. Kaplan-Meier analysis of 16 PC patients divided into high and low spike-specific IgG4 levels in cohort B (log-rank test, $p = 0.12$, median undefined vs. median 6.3). OS, overall survival. PC, pancreatic cancer.

Table S1. Cox proportional hazards analysis of factors affecting the prognosis of PC (n = 96)

	HR	95%CI	Univariate p-value	Multivariate p-value
Age ≥ 75	0.83	(0.49-1.36)	n.s.	
Sex (Female/Male)	1.15	(0.71-1.88)	n.s.	
PS ≥ 2	9.92	(4.70-20.5)	<0.001	<0.001
Jaundice (Yes/No)	2.01	(1.23-3.30)	0.006	0.011
Diabetes Mellites (Yes/No)	0.92	(0.57-1.49)	n.s.	
Location (head/body-tail)	1.39	(0.85-2.31)	n.s.	
UICC TNM classification				
T (3–4/1–2)	2.40	(1.47-3.95)	<0.001	n.s.
N (Yes/No)	1.81	(1.12-2.97)	0.017	n.s.
M (Yes/No)	4.52	(2.74-7.54)	<0.001	0.010
Surgery (Yes/No)	0.19	(0.10-0.35)	<0.001	0.011
Chemotherapy (Yes/No)	0.57	(0.34-0.97)	0.033	0.035
COVID-19 mRNA ≥ 3 vaccinations (Yes/No)	4.08	(2.25-7.59)	<0.001	<0.001
CEA > 10 (ng/mL)	2.87	(1.53-5.13)	<0.001	0.009
CA19-9 > 500 (U/mL)	2.44	(1.48-4.03)	<0.001	n.s.
NLR ≥ 2.8	2.04	(1.24-3.41)	0.006	n.s.
mGPS ≥ 1	1.67	(0.99-2.76)	0.049	n.s.
PNI ≥ 46.8	0.35	(0.20-0.58)	<0.001	n.s.
IgG4 ≥ 47.5 (mg/dL)	1.57	(0.94-2.57)	0.079	n.s.

PC, pancreatic cancer. PS, performance status. CEA, carcinoembryonic antigen. CA19-9, carbohydrate antigen 19-9. NLR, neutrophil-to-lymphocyte ratio. mGPS, modified Glasgow prognostic score. PNI, prognostic nutritional index. n.s., not significant.

Table S2. Characteristics of 66 PC patients corrected for the exclusion of surgical cases in Cohort A

	0–2 vaccinations (n=24)	≥ 3 vaccination (n=42)	p-value
Age（mean ± SD）	72.7 ± 7.4	70.5 ± 9.9	n.s.[a]
Age ≥ 75, no. (%)	8 (33.3)	17 (40.5)	n.s.[b]
Female, no. (%)	13 (54.2)	19 (45.2)	n.s.[b]
PS ≥ 2, no. (%)	4 (16.7)	5 (11.9)	n.s.[b]
Jaundice, no. (%)	10 (41.7)	18 (42.9)	n.s.[b]
Diabetes Mellites, no. (%)	12 (50.0)	24 (57.1)	n.s.[b]
Location (head), no. (%)	14 (58.3)	19 (45.2)	n.s.[b]
UICC TNM classification			
T (3–4), no. (%)	12 (50.0)	31 (73.8)	n.s.[b]
N, no. (%)	10 (41.7)	26 (61.9)	n.s.[b]
M, no. (%)	12 (50.0)	29 (69.0)	n.s.[b]
Chemotherapy, no. (%)	18 (75.0)	27 (64.3)	n.s.[b]
CEA ≥ 10, no. (%)	4 (16.7)	9 (21.4)	n.s.[b]
CA19-9 ≥ 500, no. (%)	10 (41.7)	25 (59.5)	n.s.[b]
Other factors			
NLR (mean ± SD)	3.3 ± 1.3	4.7 ± 3.4	n.s.[a]
NLR ≥ 2.8, no. (%)	12 (50.0)	31 (73.8)	n.s.[b]
mGPS (0/1/2)	17/4/3	26/10/6	n.s.[b]
mGPS ≥ 1, no. (%)	7 (29.2)	16 (38.1)	n.s.[b]
PNI (mean ± SD)	45.6 ± 5.3	44.1 ± 5.6	n.s.[a]
PNI ≥ 46.8, no. (%)	11 (45.8)	11 (26.2)	n.s.[b]
IgG4 (mean ± SD)	32.8 ± 22.2	54.5 ± 37.4	0.020[a]
IgG4 ≥ 47.5 mg/dL, no. (%)	5 (20.8)	19 (45.2)	n.s.[b]

[a] Mann–Whitney test, [b] Fisher's exact test. PC, pancreatic cancer. PS, performance status. CEA, carcinoembryonic antigen. CA19-9, carbohydrate antigen 19-9. NLR, neutrophil-to-lymphocyte ratio. mGPS, modified Glasgow prognostic score. PNI, prognostic nutritional index. n.s., not significant.

Table S3. Cox proportional hazards analysis of factors affecting PC prognosis (non surgical cases n=66)

	HR	95%CI	Univariate p-value	Multivariate p-value
Age (≥ 75)	0.91	(0.51-1.59)	n.s.	
Sex (Female/Male)	1.36	(0.79-2.37)	n.s.	
PS (≥ 2)	6.05	(2.86-12.6)	<0.001	<0.001
BMI (≥ 25)	1.14	(0.49-2.30)	n.s.	
Jaundice (Yes/No)	1.92	(1.10-3.35)	0.021	n.s.
Diabetes Mellites (Yes/No)	1.02	(0.60-1.76)	n.s.	
Location (head/body-tail)	1.65	(0.95-2.89)	n.s.	
UICC TNM classification				
T (3–4/1–2)	1.29	(0.73-2.34)	n.s.	
N (Yes/No)	1.54	(0.90-2.70)	n.s.	
M (Yes/No)	2.46	(1.39-4.52)	0.003	0.008
Chemotherapy (Yes/No)	0.38	(0.21-0.69)	0.001	0.018
COVID-19 mRNA ≥ 3 vaccinations (Yes/No)	3.99	(2.02-8.45)	<0.001	<0.001
CEA > 10 (ng/mL)	1.94	(0.98-3.60)	0.044	0.007
CA19-9 > 500 (U/mL)	1.52	(0.88-2.64)	n.s.	
NLR ≥ 2.8	1.92	(1.10-3.46)	0.024	n.s.
mGPS ≥ 1	1.42	(0.79-2.47)	n.s.	
PNI ≥ 46.8	0.56	(0.31-0.99)	0.052	n.s.
IgG4 ≥ 47.5 (mg/dL)	1.50	(0.83-2.63)	n.s.	

PC, pancreatic cancer. PS, performance status. CEA, carcinoembryonic antigen. CA19-9, carbohydrate antigen 19-9. NLR, neutrophil-to-lymphocyte ratio. mGPS, modified Glasgow prognostic score. PNI, prognostic nutritional index. n.s., not significant.

Table S4. Characteristics of high and low IgG4 groups in Cohort A

	IgG4-Low (n=61)	IgG4-High (n=35)	p-value
Age（mean ± SD）	71.4 ± 8.2	71.0 ± 8.1	n.s.[a]
Age ≥ 75, no. (%)	23 (37.7)	14 (40.0)	n.s.[b]
Female, no. (%)	35 (57.4)	15 (42.9)	n.s.[b]
PS ≥ 2, no. (%)	6 (9.8)	8 (22.9)	n.s.[b]
Jaundice, no. (%)	28 (45.9)	12 (34.3)	n.s.[b]
Diabetes Mellites, no. (%)	32 (52.5)	21 (60.0)	n.s.[b]
Location (head), no. (%)	39 (63.9)	16 (45.7)	n.s.[b]
**UICC TNM classification**			
T (3-4), no. (%)	25 (41.0)	19 (54.3)	n.s.[b]
N, no. (%)	29 (47.5)	18 (51.4)	n.s.[b]
M, no. (%)	24 (39.3)	17 (48.6)	n.s.[b]
Surgery, no. (%)	19 (31.1)	11 (31.4)	n.s.[b]
Chemotherapy, no. (%)	47 (77.0)	20 (57.1)	n.s.[b]
COVID-19 mRNA ≥ 3 vaccinations, no. (%)	26 (42.6)	23 (65.7)	0.035[b]
CEA ≥ 10, no. (%)	10 (16.4)	5 (14.3)	n.s.[b]
CA19-9 ≥ 500, no. (%)	27 (44.3)	13 (37.1)	n.s.[b]

[a] Mann–Whitney test. [b] Fisher's exact test. PC, pancreatic cancer. PS, performance status. CEA, carcinoembryonic antigen. CA19-9, carbohydrate antigen 19-9. n.s., not significant.

Table S5. Characteristics of 72 PC patients performed immunohistochemistry in Cohort A

Specimens of all primary PC	n = 72
Surgery, no. (%)	30 (41.7)
Biopsy, no. (%)	42 (58.3)
EUS-FNA, no.	39
Duodenal biopsy (invasion site), no.	2
Bile duct biopsy (invasion site), no.	1

PC, pancreatic cancer. EUS-FNA, endoscopic ultrasound-guided fine-needle aspiration biopsy.

Table S6. Characteristics of 79 patients in Cohort B

	Case (n = 79)
Age（mean±SD）	71.8 ± 10.2
Sex（Female/Male）	51/28
Disease	
Malignancy	29
PC	16
Other Malignancy	13
Benign	32
Pancreatic disease	18
Biliary disease	11
Autoimmune diseases (excluding IgG4-RD)	2
Liver dysfunction	1
IgG4-RD (Including during treatment)	18
COVID-19 mRNA ≥ 3 vaccination (Yes/No/Unknown)	57/8/14
Number of vaccination (3/4/5/6/7)	(9/8/11/24/5)

PC, pancreatic cancer. IgG4-RD, IgG4-related disease.
